# Genome-Wide Diet-Gene Interaction Analyses for Risk of Colorectal Cancer

**DOI:** 10.1371/journal.pgen.1004228

**Published:** 2014-04-17

**Authors:** Jane C. Figueiredo, Li Hsu, Carolyn M. Hutter, Yi Lin, Peter T. Campbell, John A. Baron, Sonja I. Berndt, Shuo Jiao, Graham Casey, Barbara Fortini, Andrew T. Chan, Michelle Cotterchio, Mathieu Lemire, Steven Gallinger, Tabitha A. Harrison, Loic Le Marchand, Polly A. Newcomb, Martha L. Slattery, Bette J. Caan, Christopher S. Carlson, Brent W. Zanke, Stephanie A. Rosse, Hermann Brenner, Edward L. Giovannucci, Kana Wu, Jenny Chang-Claude, Stephen J. Chanock, Keith R. Curtis, David Duggan, Jian Gong, Robert W. Haile, Richard B. Hayes, Michael Hoffmeister, John L. Hopper, Mark A. Jenkins, Laurence N. Kolonel, Conghui Qu, Anja Rudolph, Robert E. Schoen, Fredrick R. Schumacher, Daniela Seminara, Deanna L. Stelling, Stephen N. Thibodeau, Mark Thornquist, Greg S. Warnick, Brian E. Henderson, Cornelia M. Ulrich, W. James Gauderman, John D. Potter, Emily White, Ulrike Peters, on behalf of CCFR, and GECCO

**Affiliations:** 1 Keck School of Medicine, University of Southern California, Los Angeles, California, United States of America; 2 Public Health Sciences Division, Fred Hutchinson Cancer Research Center, Seattle, Washington, United States of America; 3 Division of Cancer Control and Population Sciences, National Cancer Institute, Bethesda, Maryland, United States of America; 4 Epidemiology Research Program, American Cancer Society, Atlanta, Georgia, United States of America; 5 Department of Medicine, School of Medicine, University of North Carolina, Chapel Hill, North Carolina, United States of America; 6 Division of Cancer Epidemiology and Genetics, National Cancer Institute, Bethesda, Maryland, United States of America; 7 Division of Gastroenterology, Massachusetts General Hospital and Harvard Medical School, Boston, Massachusetts; 8 Channing Division of Network Medicine, Brigham and Women's Hospital and Harvard Medical School, Boston, Massachusetts, United States of America; 9 Cancer Care Ontario, Toronto, Ontario, Canada; 10 Ontario Institute for Cancer Research, Toronto, Ontario, Canada; 11 Department of Surgery, University Health Network Toronto General Hospital, Toronto, Ontario, Canada; 12 Epidemiology Program, University of Hawaii Cancer Center, Honolulu, Hawaii, United States of America; 13 Department of Internal Medicine, University of Utah Health Sciences Center, Salt Lake City, Utah, United States of America; 14 Division of Research, Kaiser Permanente Medical Care Program, Oakland, California, United States of America; 15 Division of Hematology, Faculty of Medicine, The University of Ottawa, Ottawa, Ontario, Canada; 16 Division of Clinical Epidemiology and Aging Research, German Cancer Research Center, Heidelberg, Germany; 17 Channing Division of Network Medicine, Brigham and Women's Hospital, Boston, Massachusetts, United States of America; 18 Department of Nutrition, Harvard School of Public Health, Boston, Massachusetts, United States of America; 19 Division of Cancer Epidemiology, German Cancer Research Center, Heidelberg, Germany; 20 Translational Genomics Research Institute, Phoenix, Arizona, United States of America; 21 Stanford Cancer Institute, Palo Alto, California, United States of America; 22 Division of Epidemiology, New York University School of Medicine, New York, New York, United States of America; 23 Melbourne School of Population Health, The University of Melbourne, Melbourne, Australia; 24 Department of Medicine and Epidemiology, University of Pittsburgh Medical Center, Pittsburgh, Pennsylvania, United States of America; 25 Departments of Laboratory Medicine and Pathology and Laboratory Genetics, Mayo Clinic, Rochester, Minnesota, United States of America; 26 Division of Preventive Oncology, National Center for Tumor Diseases and German Cancer Research Center, Heidelberg, Germany; 27 Centre for Public Health Research, Massey University, Wellington, New Zealand; Dartmouth-Hitchcock Norris Cotton Cancer Center, United States of America

## Abstract

Dietary factors, including meat, fruits, vegetables and fiber, are associated with colorectal cancer; however, there is limited information as to whether these dietary factors interact with genetic variants to modify risk of colorectal cancer. We tested interactions between these dietary factors and approximately 2.7 million genetic variants for colorectal cancer risk among 9,287 cases and 9,117 controls from ten studies. We used logistic regression to investigate multiplicative gene-diet interactions, as well as our recently developed Cocktail method that involves a screening step based on marginal associations and gene-diet correlations and a testing step for multiplicative interactions, while correcting for multiple testing using weighted hypothesis testing. Per quartile increment in the intake of red and processed meat were associated with statistically significant increased risks of colorectal cancer and vegetable, fruit and fiber intake with lower risks. From the case-control analysis, we detected a significant interaction between rs4143094 (10p14/near *GATA3*) and processed meat consumption (OR = 1.17; p = 8.7E-09), which was consistently observed across studies (p heterogeneity = 0.78). The risk of colorectal cancer associated with processed meat was increased among individuals with the rs4143094-TG and -TT genotypes (OR = 1.20 and OR = 1.39, respectively) and null among those with the GG genotype (OR = 1.03). Our results identify a novel gene-diet interaction with processed meat for colorectal cancer, highlighting that diet may modify the effect of genetic variants on disease risk, which may have important implications for prevention.

## Author Summary

High intake of red and processed meat and low intake of fruits, vegetables and fiber are associated with a higher risk of colorectal cancer. We investigate if the effect of these dietary factors on colorectal cancer risk is modified by common genetic variants across the genome (total of about 2.7 million genetic variants), also known as gene-diet interactions. We included over 9,000 colorectal cancer cases and 9,000 controls that were not diagnosed with colorectal cancer. Our results provide strong evidence for a gene-diet interaction and colorectal cancer risk between a genetic variant (rs4143094) on chromosome 10p14 near the gene *GATA3* and processed meat consumption (p = 8.7E-09). This genetic locus may have interesting biological significance given its location in the genome. Our results suggest that genetic variants may interact with diet and in combination affect colorectal cancer risk, which may have important implications for personalized cancer care and provide novel insights into prevention strategies.

## Introduction

Colorectal cancer is the third most common neoplasm and the third leading cause of cancer death in both men and women across most ethnic-racial groups [1]. Intake of various dietary factors, most notably, meat, fruits/vegetables, and fiber, have been extensively investigated in relation to colorectal cancer risk. Overall, the evidence suggests that consumption of red and processed meat modestly increase the risk of colorectal cancer [2], [3]; and fruits [4], vegetables [4], [5], and fiber [6]–[8] decrease risk, although these associations have not been observed across all studies [2], [9], [10], perhaps due to methodological differences and unaccounted modifying effects.

More recently, studies have focused on the potential modifying effects of common genetic variants, single nucleotide polymorphisms (SNPs), on the relationship between dietary factors and risk of colorectal cancer. However, attention has largely focused on candidate SNPs in genes directly involved in the metabolism of selected nutrients; for example, metabolism of B-vitamins [11], key nutrients found in fruits and vegetables; or the metabolism of carcinogenic by-products resulting from cooking or processing of meat [12]. From these candidate gene/pathway-approaches, few genetic variants have been consistently identified and further investigation is warranted.

Large datasets from genome-wide association studies of colorectal cancer are now available for a comprehensive analysis of gene-diet interactions on the risk of colorectal cancer. To date, one genome-wide study of gene-diet interactions focusing on microsatellite stable/microsatellite-instability low colorectal cancer (1,191 cases, 990 controls) reported no statistically significant gene-diet interactions after replication in an independent dataset [13]. The authors highlighted the need for collaborative consortia to increase sample size, with central quality control procedures and careful standardization and harmonization of definitions and measurements. Hutter et al., using data from the Genetics and Epidemiology of Colorectal Cancer Consortium (GECCO) on 7,106 colorectal cancer cases and 9,723 controls from 9 studies focused on 10 previously identified colorectal cancer-susceptibility loci and conducted a systematic search for interaction with selected lifestyle and dietary factors. The strongest statistical evidence was observed for interaction for vegetable consumption and rs16892766, located on chromosome 8q23.3 near the *EIF3H* and *UTP23* genes (p = 1.3E-04) [14].

In this large combined analysis using GECCO from 10 case-control and nested cohort studies comprising 9,287 colorectal cancer cases and 9,120 controls, we build upon these previous reports [13], [14] to examine over 2.7 million common polymorphisms for multiplicative interactions with selected dietary factors (red meat, processed meat, fiber, fruit and vegetables) and risk of colorectal cancer. For our primary analyses we used conventional case-control logistic regression that included an interaction term as well as our recently developed Cocktail method, which integrates several novel GxE methods to improve statistical power under various scenarios [15].

## Results

**Figure 1:**
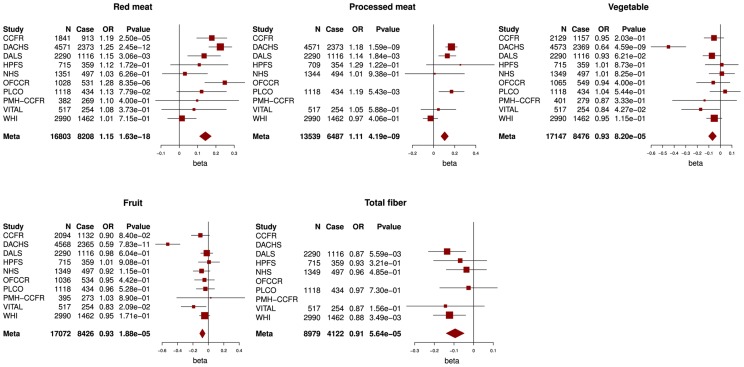
Associations between red and processed meat, vegetable, fruit and fiber intake and colorectal cancer risk. Odds ratios (ORs) per quartile of increasing intake, lowest quartile = reference group, N = total number of subjects, case = number of cases.

Characteristics of the 10 studies are described in Table S1. Mean intake and quartile cut points of each dietary factor per study are provided in Table S2 and S3. Across all studies we observed an increase in colorectal cancer risk for red meat consumption (OR_per quartile_ = 1.15,p = 1.6E-18) and processed meat consumption (OR_per quartile_ = 1.11,p = 4.2E-09). Decreased colorectal cancer risk was observed for vegetable intake (OR_per quartile_ = 0.93, p = 8.2E-05), fruit intake (OR_per quartile_ = 0.93, p = 1.9E-05) and fiber intake (OR_per quartile_ = 0.91, p = 5.6E-05, Figure 1).

**Figure 2:**
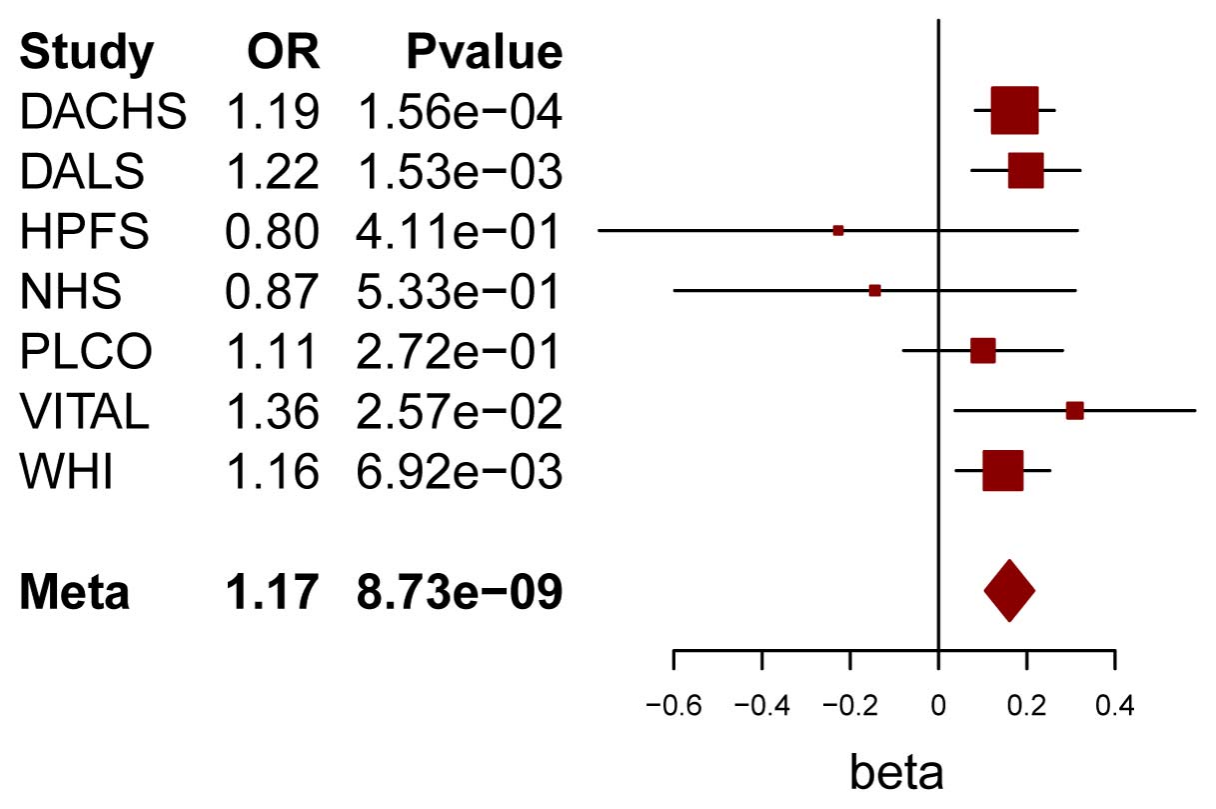
Forest plot for meta-analysis of interaction analysis for rs4143094 and processed meat. Odds ratios (ORs) and 95% confidence intervals (95% CI) are presented for each additional copy of the count (or tested) allele (T) and for each increasing quartile of processed meat intake in the multiplicative interaction model. The box sizes are proportional in size to the inverse of the variance for each study, and the lines visually depict the confidence interval. Results from the fixed-effects meta-analysis are shown as diamonds. The width of the diamond represents the confidence interval.

**Figure 3:**
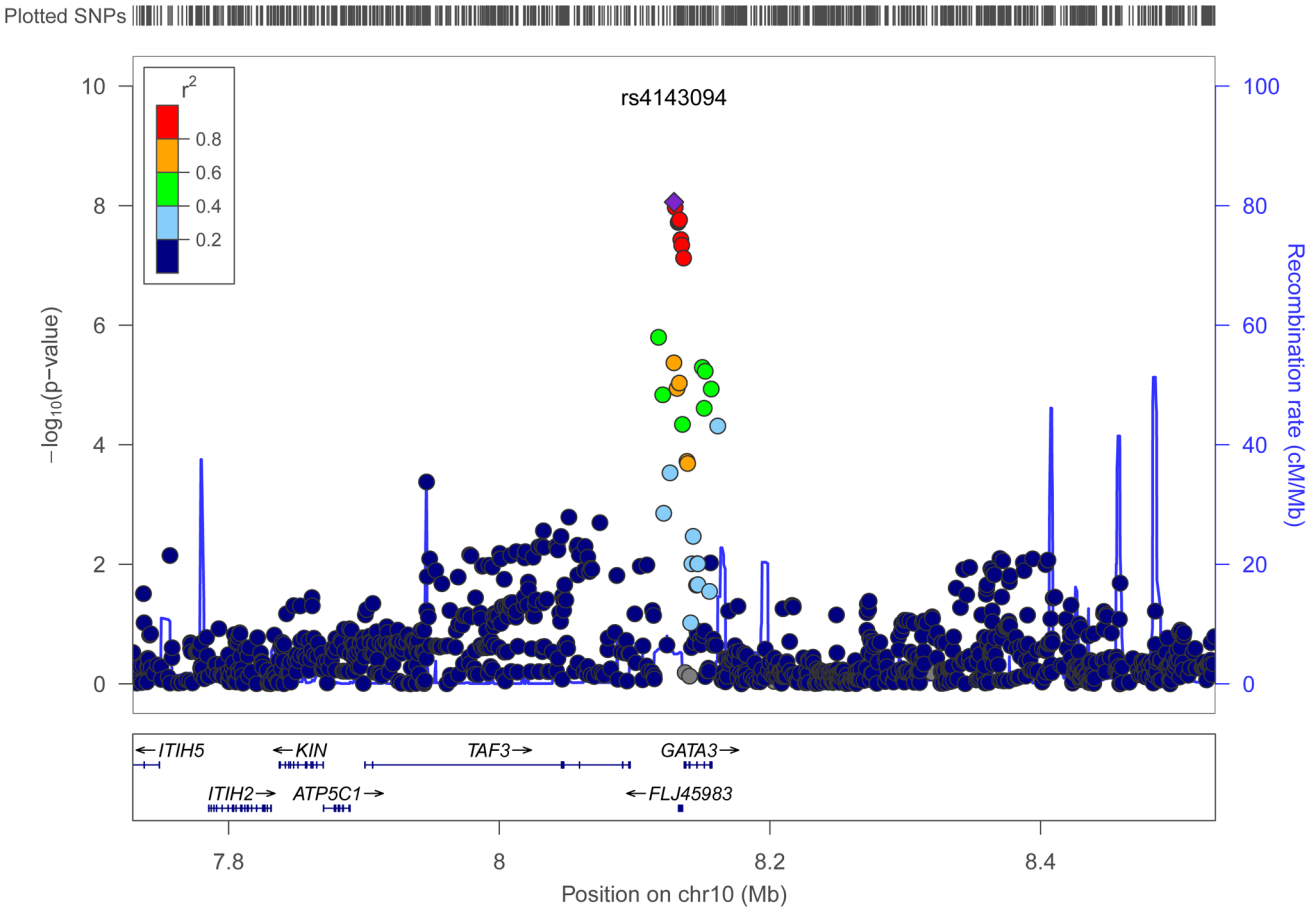
Regional association results for the interaction between processed meat and rs4143094 with surrounding SNPs. The top half of the figure has physical position along the x-axis, and the −log10 of the meta-analysis p-value of the interaction term on the y-axis. Each dot on the plot represents the p-value of the interaction for one SNPxD in relation to colorectal cancer conducted across all studies. The most significant SNP in the region (index SNP) is marked as a purple diamond. The color scheme represents the pairwise correlation (r^2^) for the SNPs across the region with the index SNP. Correlation was calculated using the HapMap CEU data. The bottom half of the figure shows the position of the genes across the region. These regional association plots are also known as LocusZoom plots.

Using conventional case-control logistic regression to test for multiplicative interactions we identified a genome-wide significant interaction between variants at chromosome 10p14 and processed meat (Table 1). Within the 10p14 region rs4143094 showed the most significant interaction with processed meat (OR_interaction_ for each copy of T-allele and increasing quartile of processed meat = 1.17, p = 8.73E-09, Table 1 and Figure 2), with no evidence of heterogeneity (p_heterogeneity_ = 0.78). This SNP (rs4143094), as well as correlated SNPs surrounding the rs4143094 SNP, indicate a strong signal peak in the 10p14 region near the *GATA3* gene; as expected SNPs less correlated with rs4143094 show less significant interactions (Figure 3). Stratified by genotype, the risk for colorectal cancer associated with each increasing quartile of processed meat was increased in individuals with the rs4143094-TG and -TT genotypes (OR = 1.20, 95% CI = 1.13–1.26 and OR = 1.39, 95% CI = 1.22–1.59, respectively) and null in individuals with the rs4143096-GG genotype (OR = 1.03, 95% CI = 0.98–1.07, Table 2). Results are very similar for minimal and multivariable adjusted ORs. In addition, the stratified results Table S4 show interaction results using one common reference group. This common SNP (average allele frequency of T allele = 0.25) was directly genotyped in most studies or imputed with high accuracy (imputation r^2^>0.89). With the other dietary factors evaluated, no interactions using the conventional case-control logistic regression analysis reached the genome-wide significance threshold (Table S5).

**Table 1:** Top three SNPs according to lowest p-value for interactions with processed meat for risk of colorectal cancer using conventional case-control logistic regression approach.

SNP	Chr	Position	Context	Gene	CountAllele	CAF*	OR_interaction_ **	95% CI	p_interaction_	p_heterogeneity_
rs4143094	10p14	8129142	promoter	*GATA3*	T	0.21–0.27	1.17	1.11–1.23	8.73E-09	0.78
			intergenic	*GATA3-AS1*						
rs485411	10p14	8133191	promoter	*GATA3*	C	0.20–0.27	1.18	1.11–1.25	1.72E-08	0.70
			non-coding transcript variant	*GATA3-AS1*						
rs1269486	10p14	8136205	promoter	*GATA3*	A	0.22–0.26	1.18	1.11–1.25	7.53E-08	0.65

* CAF, count allele frequency. Count (or tested) allele is defined as the allele that was coded as 1 in the logistic regression (the other allele was coded as 0).

** interaction OR for each copy of the count allele and for each increasing quartile of processed meat intake.

**Table 2:** Association of processed meat and risk of colorectal cancer by genotype strata for rs4143094.

Adjustment factors	rs4143094	N Case	N Control	Association per quartile of processed meat intake		
				OR	95% CI	P value
Minimal*	GG	3627	3986	1.03	0.98–1.07	0.28
	TG	2428	2610	1.2	1.13–1.26	2.70E-10
	TT	430	445	1.39	1.22–1.59	1.10E-06
Multivariable**	GG	3542	3887	0.98	0.93–1.03	0.5
	TG	2375	2547	1.14	1.08–1.22	1.18E-05
	TT	418	439	1.36	1.18–1.56	1.35E-05

*Minimal adjusted models included age, sex, study site, energy and PCs.

**Multivariable adjusted models additionally included: BMI, smoking, alcohol and other dietary factors.

Multivariable-adjusted analysis is limited to samples with available data for all covariates used in the analysis.

With the other dietary factors, no interactions with any of the 2.7M SNPs were statistically significant using the conventional logistic regression analysis. Furthermore, we did not observe any novel interactions using our Cocktail method or the two exploratory statistical methods by Gauderman et al. [16] and Dai et al. [17] (data not shown).

## Discussion

Genome-wide scans have successfully identified numerous risk loci for colorectal cancer; consortia pooling multiple studies for increased statistical power have continued to identify additional susceptibility loci [18]–[24]. However, only limited work has been pursued at a genome-wide scale to identify gene-diet interactions. Using individual-level data from ten studies with harmonized dietary intake variables on a total of over 9,000 cases and 9,000 controls, we have conducted a genome-wide analysis for GxE interactions. Using conventional statistical methods, as well as our novel method aiming to improve statistical power, we provide evidence for a novel interaction between rs4143094 and processed meat intake.

The variants in the 10p14 region interacting with processed meat consumption reside within and upstream of GATA binding protein 3 (*GATA3*) gene. *GATA3* has long been associated with T cell development, specifically Th2 cell differentiation [25]. *GATA3* is up-regulated in ulcerative colitis [26], which is associated with increased risk of colorectal cancer [27]. However, the role of *GATA* genes as transcription factors extends to epithelial structures with a known role in breast, prostate and other cancers [28]–[30]. GATA factors are involved in cellular maturation with proliferation arrest and cell survival. Loss of GATA genes or silencing of expression have been described for breast, colorectal and lung cancers [30].

To further explore this locus, we evaluated the potential functional impact of the most significant SNP in this locus as well as correlated SNPs querying multiple bioinformatics databases, such as Encode and NIH Roadmap (Table S6). The most significant SNP rs4143094 is about 7.2 kb upstream of GATA and resides in a 9.5 kb LD block (r^2^>0.8) containing 19 highly correlated SNPs, including rs1269486, which shows the third most significant interaction in this region (Table 1). The rs1269486 variant is located 1420 bases upstream of *GATA3* in a region of open chromatin (DNase I hypersensitivity) with histone methylation patterns consistent with promoter activity in a colorectal cancer cell line (CACO2; Figure S1). As would be expected of a promoter region, experimental evidence supports Pol2 binding along with the transcription factors c-Fos, JunD, and c-Jun [31]. Many of the other SNPs upstream of *GATA3* are located in *GATA3*-antisense RNA1 (*GATA3*-*AS1*) (formerly FLJ45983). *GATA3*-*AS1* is a non-coding RNA that may regulate *GATA3* transcript levels in the cell. Further studies are required to elucidate the relationship between *GATA3* and *GATA3*-*AS1* and determine whether variants in the 10p14 region cause perturbations in regulation.

A plausible though speculative biological basis for our findings is that processed meat triggers a pro-tumorigenic inflammatory or immunological response [32] that may necessitate proper GATA3 transcription levels. Nonetheless, the precise mechanism by which deregulation of *GATA3* is linked to colorectal cancer upon consumption of high levels of processed meat remains unclear. Further study of the role of variants in *GATA3* in colorectal cancer will yield more insight into their functional significance.

The interaction between variants in locus 10p14 and processed meat were identified by the conventional case-control logistic regression analysis. This locus was not identified through our Cocktail method or any of the other exploratory methods (Text S2). However, this is not surprising given that the SNPs in this locus are not strongly associated with colorectal cancer (p = 0.26 for rs4143094) and not strongly correlated with processed meat (p = 0.25 for rs4143094) and, accordingly, SNPs in this locus were not prioritized in the Cocktail analysis. However, we were somewhat surprised to not identify additional interactions with any of the dietary factors using our Cocktail method, given the expected improvement in power under various scenarios. We recognize that the field of GxE analyses is at an early stage compared with studies for marginal gene-diseases associations. It will be important to see more large-scale empirical GxE studies to judge the impact and potential power gain of the novel GxE methods.

Our analysis has some limitations and notable strengths. We adopted a flexible approach to data harmonization of dietary factors, in a similar fashion to those proposed by other projects [33], [34]. We focused on dietary variables that were collected in a similar manner and allowed for harmonization across a large subset of the studies. Ideally, our findings will be replicated in other populations. While a substantial larger number of GWAS have been conducted for colorectal cancer, limited studies have collected information on processed meat and other dietary variables. In the present study, we did not divide our large sample into discovery and replication sets, as it has been shown that the most powerful analytical approach is a combined analysis across all studies [35]. This approach is increasingly used as more samples with GWAS data are becoming available [36]. Importantly, we observed no evidence of heterogeneity in the estimates by study, which suggests that results are consistent across studies.

We not only used the conventional case-control logistic regression, but also took advantage of our recently developed Cocktail method as a second primary analysis approach to potentially improve statistical power. We note that even though for the Cocktail method different interaction tests (case-only and case-control) were used depending on the screening step, the overall genome-wide type I error is controlled at 0.05 (genome-wide level of α was set to 5E-08), just like the conventional case-control method. As we investigated five dietary factors and used two primary methods additional adjustment for multiple comparisons may be warranted. However, we want to point out that the dietary variables were correlated, e.g. correlation between fruits and vegetables was 0.38, between fruits and fiber was 0.52 or between red and processed meat was 0.62 adjustments for these not independent test is less straight forward. Similarly, the primary methods are not independent from each other, for instance the testing step of the Cocktail method used the case-control or case-only testing, which are consistent or correlated with the conventional case-control analysis. Accordingly, additional multiple comparison adjustment for 5 variables and 2 tests would be too conservative, nevertheless our interaction finding for 10p14 and processed meat would likely remain marginally significant.

With the investment of large GWAS consortium built on well-characterized studies, we are now well-positioned to identify potential interactions between genetic loci and environmental risk factors with respect to colorectal cancer risk. In this study, we have identified a novel interaction between rs4143094 and processed meat. This genetic locus may have interesting biological significance given its proximity to genes plausibly associated with pathways relevant to colorectal carcinogenesis. Nonetheless, further functional analysis is required to uncover the specific mechanisms by which this genetic locus modulates the association between intake of processed meat and colorectal cancer risk.

## Materials and Methods

### Study participants

This analysis uses data from the Colon Cancer Family Registry (CCFR) and the Genetics and Epidemiology of Colorectal Cancer Consortium (GECCO, Text S1 and Table S1) as described previously [14], [37]. All cases were defined as colorectal adenocarcinoma and confirmed by medical records, pathologic reports, or death certificate. All studies received ethical approval by their respective Institutional Review Boards and participants gave written informed consent.

### Genotyping, quality assurance/quality control and imputation

Average sample and SNP call rates, and concordance rates for blinded duplicates have been previously published [37]. In brief, genotyped SNPs were excluded based on call rate (<98%), lack of Hardy-Weinberg Equilibrium in controls (HWE, p<1×10^−4^), and low minor allele frequency (MAF). We imputed the autosomal SNPs of all studies to the CEU population in HapMap II. SNPs were restricted based on per-study minor allele count >5 and imputation accuracy (R^2^>0.3) to avoid missing any interactions. After imputation and quality control (QC) analyses, approximately 2.7M SNPs were used in the analysis.

All analyses were restricted to individuals of European ancestry, defined as samples clustering with the Utah residents with Northern and Western European ancestry from the CEPH collection (CEU) population in principal component analysis [38], including the HapMap II populations as reference.

### Harmonization of dietary factors

Information on basic demographics and environmental risk factors was collected by using in-person interviews and/or structured questionnaires, as detailed previously [39]–[48]. The multi-step data harmonization procedure applied in this study is described in detail by Hutter et al. [14]. Here we focus on selected dietary variables for intake of red and processed meat, fruits, vegetables (all measured in servings per day) and fiber (measured as g/day). These variables were coded as sex- and study-specific quartiles, where the quartile groups were coded 1 to 4 of the quartile within the controls of each study and sex. For studies that due to limited number of questions assessed dietary intake in categories rather than as continuous variables and had less than 4 intake categories, we assigned these categories to the 2^nd^ and 3^rd^ or 1st to 3^rd^ quartile, as appropriate. The lowest category of exposure was used as the reference and each dietary factor was analyzed as an ordinal variable (e.g., 1, 2, 3, 4) in the model. Data harmonization was performed using SAS and T-SQL.

### Statistical methods

Statistical analyses of all samples were conducted centrally at the GECCO coordinating center on individual-level data to ensure a consistent analytical approach. Unless otherwise indicated, we adjusted for age at the reference time, sex (when appropriate), center (when appropriate), total energy consumption (if available) and the first three principal components from EIGENSTRAT to account for potential population substructure. The dietary variables were coded as described above. Each directly genotyped SNP was coded as 0, 1, or 2 copies of the variant allele. For imputed SNPs, we used the expected number of copies of the variant allele (the “dosage”), which has been shown to give unbiased test statistics [49]. Genotypes were treated as continuous variables (i.e. log-additive effects). Each study was analyzed separately using logistic regression models and study-specific results were combined using fixed-effects meta-analysis methods to obtain summary odds ratios (ORs) and 95% confidence intervals (CIs) across studies. We calculated the heterogeneity p-values by Woolf's test [50]. Quantile-quantile (Q-Q) plots were assessed to determine whether the distribution of the p-values was consistent with the null distribution (except for the extreme tail).

To test for interactions between SNPs and dietary risk factors, we conduct two primary analyses: 1) conventional case-control logistic regression analysis including a multiplicative interaction term; 2)our newly developed Cocktail method [15]. For the conventional logistic regression analysis, we modeled the SNP by environment (GxE) interaction by the product of the SNP and the dietary variable (which is in this study the E), adjusting for age, sex, study site, energy, principal components and the main effects of the SNP and dietary variable. Adjustment for additional variables, smoking, alcohol, BMI and other dietary variables did not appreciably change the results. A two-sided p-value of 5×10^−8^ for a SNP-diet factor interaction was considered statistically significant, yielding a genome-wide significance level 0.05 assuming about 1 million independent tests across the genome (0.05/1,000,000 = 5×10^−8^) [51]–[56].

Motivated by recent advances in methods development for detecting GxE interaction [17], [57]–[60], our second approach was based on our recently developed Cocktail method. This statistical method combines the most appealing aspect of several newly developed GxE methods with the goal of creating a comprehensive and powerful test for genome-wide detection of GxE [15]. In brief, this method consists of two-steps: a screening step to prioritize SNPs and a testing step for GxE interaction. Specifically, for the screening step, we ranked and prioritized variants through a genome-wide screen of each of the 2.7M SNPs (referred to as “G”) by the maximum of the test statistics from marginal association of Gs on disease risk [58], and correlation between G and environmental/dietary variable (E) in cases and controls combined [59], a combination which allows for identifying variants with different interaction patterns.

Based on the ranks of these SNPs from screening, we used a weighted hypothesis framework to partition SNPs into groups with higher ranked groups having less stringent alpha-level cut-offs for interaction [60], [61]. We followed the grouping scheme used by Ionita et al. [61] such that for example, the first 3 groups consist of 5 SNPs (SNP 1 to 5), 10 SNPs (SNP 6 to 15) and 20 SNPs (SNP 16 to 36), and the corresponding cut-offs are α_group 1_ = α/(2*5) = 0.005, α_group 2_ = α/(4*10) = 0.00125 and α_group 3_ = α/(8*20) = 0.0003, respectively, so on and so forth, to maintain the overall genome-wide alpha level of 0.05. To avoid testing correlated SNPs, we pruned SNPs based on proximity (exclude any SNP within +/−50 kb of the selected SNP) given that LD pruning is difficult to implement for large number of SNPs. While the choice of the group size is arbitrary our simulation study showed that different group size did not impact the results substantially, and importantly, we chose the group size before looking at the results.

The second step of the Cocktail method is the testing step. We tested each of the G's for GxE interactions using the case-only (CO) logistic regression test. The use of the CO test is justified because we did not observe correlation between G and any of the tested dietary factors, and it has been shown that under the independence assumption the CO test provides substantial efficiency gain over the conventional CC test [62]. Since the CO is not independent of the correlation screening (a requirement to avoid inflation of type I error rates) [63], we used CO test only when the maximum screening test statistic came from the marginal association, and the case-control test otherwise.

In Text S2, we describe two secondary statistical GxE methods that we used to explore other novel GxE methods: the 2-step method by Gauderman et al. method [16] and a 2 degree of freedom joint test for marginal associations of G and GxE interaction by Dai et al. [17]. All analyses were conducted using the R programming language [64].

## Supporting Information

Figure S1 Functional annotation of rs4143094 and correlated SNPs in chromosome 10.
(PDF)

Table S1 Descriptive characteristics of each study.
(DOCX)

Table S2 Mean intake of red meat, processed meat, vegetable, fruit and fiber intake by study.
(DOCX)

Table S3 Quartile cut points for intake of red meat, processed meat, vegetable, fruit and fiber intake by study and sex.
(DOCX)

Table S4 Interaction between rs4143094 and processed meat intake for risk of colorectal cancer based on one common reference group and stratified analysis by genotype (last row) and by quartiles of processed meat (last column).
(DOCX)

Table S5 Top three most significant GxE interactions for red meat, vegetable, fruit and fiber using conventional case-control logistic regression analyses (for regions with multiple highly correlated SNPs only the most significant SNP was included).
(DOCX)

Table S6 Description of bioinformatics tools used for functional follow-up of non-coding regions.
(DOCX)

Text S1 Study populations. Description of the methodology and individual study populations included in this meta-analysis.
(DOCX)

Text S2 Additional statistical analysis. Description of the additional statistical methods used in this meta-analysis.
(DOCX)

Text S3 Functional annotation of identified loci. Description of the methodology for functionally annotating significant loci.
(DOCX)

Text S4 References supplementary text.
(DOCX)
