# Supplementary figures and images for: Genome-Wide Diet-Gene Interaction Analyses for Risk of Colorectal Cancer
Source: PLoS Genet. 2014 Apr 17;10(4):e1004228. doi: 10.1371/journal.pgen.1004228 (PMC3990510; doi:10.1371/journal.pgen.1004228)

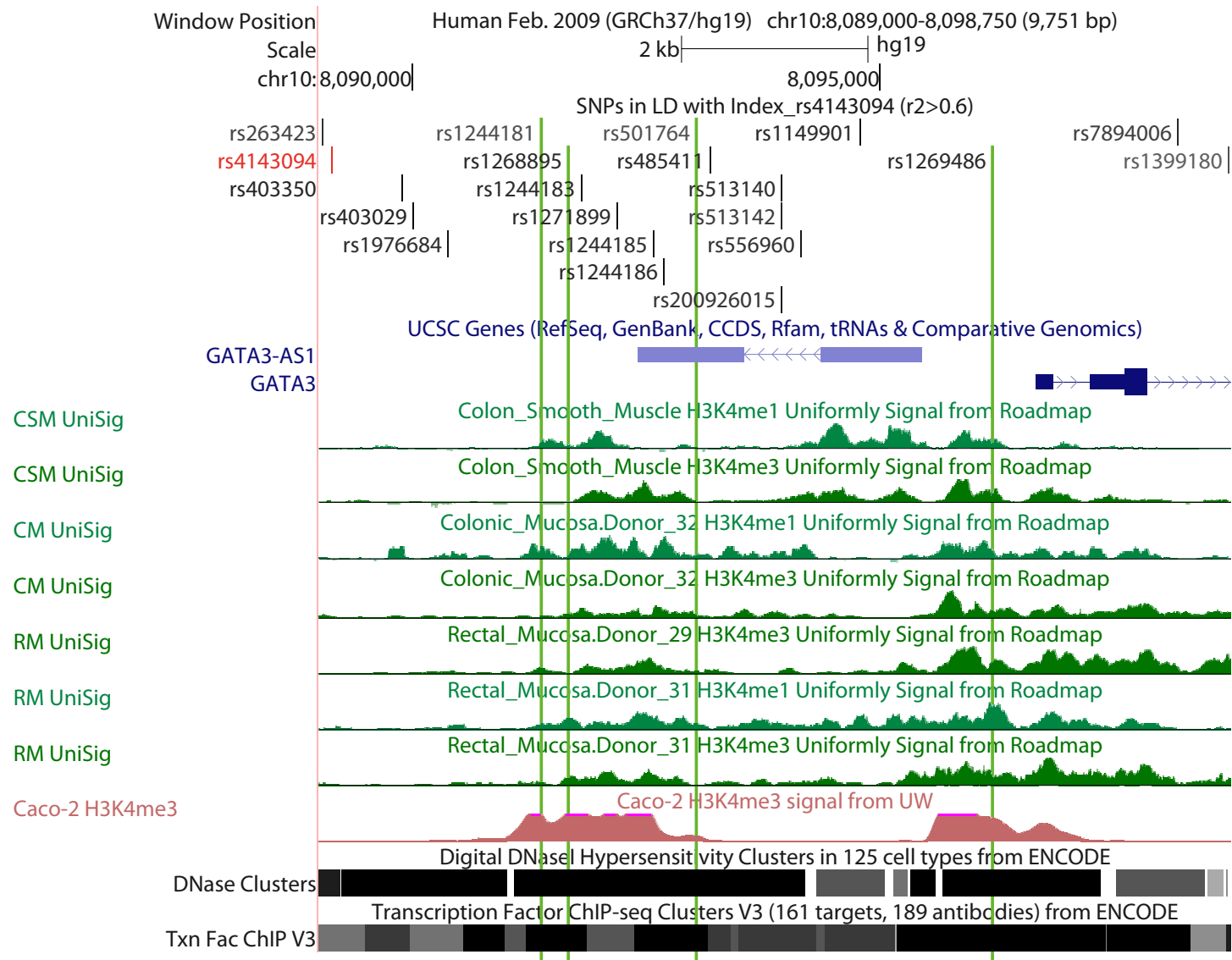

Supplement: Figure S1 — Functional annotation of rs4143094 and correlated SNPs in chromosome 10. (PDF) [file pgen.1004228.s001.pdf]
