# Supplementary material for: Genome-Wide Diet-Gene Interaction Analyses for Risk of Colorectal Cancer
Source: PLoS Genet. 2014 Apr 17;10(4):e1004228. doi: 10.1371/journal.pgen.1004228 (PMC3990510; doi:10.1371/journal.pgen.1004228)
Supplement: Table S1 — Descriptive characteristics of each study. (DOCX) [file pgen.1004228.s002.docx]

**Table S1. Descriptive characteristics of each study**

| **Study Name** | **Other Name** | **Design** | **Country** | **Cases*** | **Controls*** | **Age range (yrs)** | **Mean Age (yrs)** | **Female (%)** |
| --- | --- | --- | --- | --- | --- | --- | --- | --- |
| Colon Cancer Family Registry° | CCFR | case-control | U.S., Canada, Australia | 1,171 | 980 | 20-81 | 54.2 | 50 |
| Darmkrebs: Chancen der Verhütung durch Screening | DACHS | case-control | Germany | 2,376 | 2,206 | 33-99 | 68.7 | 39.9 |
| Diet, Activity and Lifestyle Study | DALS | case-control | U.S. | 1,116 | 1,174 | 28-79 | 63.9 | 44.9 |
| Health Professionals Follow-up Study | HPFS | cohort | U.S. | 403 | 402 | 48-83 | 65.2 | 0 |
| Nurses’ Health Study | NHS | cohort | U.S. | 549 | 955 | 44-69 | 59.8 | 100 |
| Ontario Familial Colorectal Cancer Registry | OFCCR | case-control | Canada | 650 | 522 | 29-77 | 62 | 52 |
| Postmenopausal Hormone study- Colon Cancer Family Registry | PMH-CCFR | case-control | U.S. | 280 | 122 | 48-73 | 62.8 | 100 |
| Prostate, Lung, Colorectal and Ovarian Cancer Screening Trial | PLCO | cohort | U.S. | 991 | 937 | 55-75 | 64.3 | 41.6 |
| VITamins And Lifestyle | VITAL | cohort | U.S. | 285 | 288 | 50-76 | 66.5 | 47.6 |
| Women’s Health Initiative | WHI | cohort | U.S. | 1,466 | 1,531 | 50-79 | 66.3 | 100 |

* Sample numbers based on genotype data and not all individuals have dietary information. Numbers varies for specific dietary variables of interest due to missing dietary data.

°CCFR is a collaborating study with GECCO.
