# Supplementary material for: Genome-Wide Diet-Gene Interaction Analyses for Risk of Colorectal Cancer
Source: PLoS Genet. 2014 Apr 17;10(4):e1004228. doi: 10.1371/journal.pgen.1004228 (PMC3990510; doi:10.1371/journal.pgen.1004228)
Supplement: Table S2 — Mean intake of red meat, processed meat, vegetable, fruit and fiber intake by study. (DOCX) [file pgen.1004228.s003.docx]

**Table S2: Mean intake of red meat, processed meat, vegetable, fruit and fiber intake by study**

| Study | **Red meat (servings/day)** | **Processed meat (servings/day)** | **Vegetable intake (servings/day)** | **Fruit intake (servings/day)** | **Fiber intake**  **(g/day)** |
| --- | --- | --- | --- | --- | --- |
|  | **Mean (SD)** | **Mean (SD)** | **Mean (SD)** | **Mean (SD)** | **Mean (SD)** |
| CCFR | 0.61 (0.58) | NA | 2.04 (1.48) | 1.55 (1.19) | NA |
| DACHS | 0.74 (0.35) | 0.80 (0.43) | 1.15 (0.35) | 0.99 (0.55) | NA |
| DALS | 1.01 (0.80) | 0.23 (0.26) | 3.39 (2.25) | 2.21 (1.76) | 24.59 (10.70) |
| HPFS | 0.79 (0.69) | 0.07 (0.10) | 3.63 (1.95) | 2.70 (1.70) | 23.03 (7.15) |
| NHS | 0.67 (0.56) | 0.09 (0.18) | 3.60 (1.82) | 2.53 (1.50) | 18.73 (5.78) |
| OFCCR | 0.60 (0.53) | NA | 2.05 (1.24) | 1.66 (1.13) | NA |
| PLCO | 1.14 (0.92) | 0.26 (0.29) | 5.11 (2.28) | 3.38 (2.12) | 23.40 (9.14) |
| PMH-CCFR | 0.43 (0.33) | NA | 1.63 (0.98) | 1.41 (1.00) | NA |
| VITAL | 0.66 (0.53) | 0.29 (0.33) | 2.07 (1.18) | 1.91 (1.44) | 18.49 (8.49) |
| WHI | 0.69 (0.56) | 0.27 (0.32) | 2.24 (1.25) | 1.91 (1.21) | 16.08 (6.94) |
| All | 0.76 (0.67) | 0.41 (0.44) | 2.40 (1.85) | 1.81 (1.46) | 20.25 (9.04) |

NA, not available as the variable was not assessed in the study
