# Supplementary material for: Genome-Wide Diet-Gene Interaction Analyses for Risk of Colorectal Cancer
Source: PLoS Genet. 2014 Apr 17;10(4):e1004228. doi: 10.1371/journal.pgen.1004228 (PMC3990510; doi:10.1371/journal.pgen.1004228)
Supplement: Table S3 — Quartile cut points for intake of red meat, processed meat, vegetable, fruit and fiber intake by study and sex. (DOCX) [file pgen.1004228.s004.docx]

**Table S3: Quartile cut points for intake of red meat, processed meat, vegetable, fruit and fiber intake by study and sex**

|  | **Male** | | | | **Female** | | | |
| --- | --- | --- | --- | --- | --- | --- | --- | --- |
|  | **Quartile 1** | **Quartile 2** | **Quartile 3** | **Quartile 4** | **Quartile 1** | **Quartile 2** | **Quartile 3** | **Quartile 4** |
| **Red Meat** | | | | | | | | |
| CCFR | 0.03--0.29 | 0.29--0.43 | 0.43--0.71 | 0.71--8 | 0.03--0.29 | 0.29--0.43 | 0.43--0.57 | 0.57--4 |
| OFCCR | 0.03--0.29 | 0.29--0.43 | 0.43--0.71 | 0.71--5 | 0.03--0.29 | 0.29--0.43 | 0.43--0.57 | 0.57--6 |
| DALS | 0--0.575 | 0.575--0.95 | 0.95--1.52 | 1.52--8 | 0--0.34 | 0.34--0.61 | 0.61--0.94 | 0.94--5.59 |
| PLCO | 0--0.71 | 0.71--1.085 | 1.085--1.73 | 1.73--7.56 | 0.03--0.37 | 0.37--0.61 | 0.61--1.0075 | 1.0075--3.96 |
| WHI | NA | NA | NA | NA | 0--0.3075 | 0.3075--0.55 | 0.55--0.92 | 0.92--6.12 |
| DACHS | 0--0.63 | 0.63--0.84 | 0.84--1.06 | 1.06--3 | 0--0.35 | 0.35--0.63 | 0.63--0.84 | 0.84--3 |
| HPFS | 0--0.2225 | 0.2225--0.62 | 0.62--1.05 | 1.05--6.36 | NA | NA | NA | NA |
| NHS | NA | NA | NA | NA | 0--0.28 | 0.28--0.56 | 0.56--0.94 | 0.94--4.89 |
| VITAL | 0--0.32 | 0.32--0.615 | 0.615--1.0125 | 1.0125--4.96 | 0--0.2175 | 0.2175--0.46 | 0.46--0.7425 | 0.7425--3.18 |
| PMH-CCFR | NA | NA | NA | NA | 0.03--0.14 | 0.14--0.43 | 0.43--0.57 | 0.57—4 |
| **Processed Meat** | | | | | | | | |
| DALS | 0--0.08 | 0.08--0.2 | 0.2--0.37 | 0.37--3.89 | 0--0.04 | 0.04--0.1 | 0.1--0.2 | 0.2--1.15 |
| PLCO | 0--0.11 | 0.11--0.22 | 0.22--0.4 | 0.4--2.11 | 0--0.05 | 0.05--0.09 | 0.09--0.18 | 0.18--1.11 |
| WHI | NA | NA | NA | NA | 0--0.07 | 0.07--0.17 | 0.17--0.37 | 0.37—4 |
| DACHS | 0--0.63 | 0.63--0.84 | 0.84--1.07 | 1.07--3 | 0--0.28 | 0.28--0.63 | 0.63--0.84 | 0.84—3 |
| HPFS | NA | 0--0.04 | 0.04--0.5 | NA | NA | NA | NA | NA |
| NHS | NA | NA | NA | NA | NA | 0--0.07 | 0.07--2.5 | NA |
| VITAL | 0--0.09 | 0.09--0.26 | 0.26--0.4925 | 0.4925--2.22 | 0--0.04 | 0.04--0.13 | 0.13--0.28 | 0.28--3.5 |
| **Vegetable** | | | | | | | | |
| CCFR | 0.03--1 | 1--1.215 | 1.215--2 | 2--13 | 0.03--1 | 1--2 | 2--3 | 3—15 |
| OFCCR | NA | 0.07--1 | 1--6 | NA | 0.07--1 | 1--2 | 2--3 | 3—10 |
| DALS | 0.13--1.94 | 1.94--2.91 | 2.91--4.255 | 4.255--20 | 0.05--1.89 | 1.89--2.9 | 2.9--4.16 | 4.16--20 |
| PLCO | 0.79--3.79 | 3.79--5.05 | 5.05--6.4875 | 6.4875--20 | 0.88--3.3625 | 3.3625--4.445 | 4.445--5.7025 | 5.7025--13.85 |
| WHI | NA | NA | NA | NA | 0.03--1.38 | 1.38--2.04 | 2.04--2.945 | 2.945--7.58 |
| DACHS | NA | 0--1.12 | 1.12--2.56 | NA | NA | 0--1.12 | 1.12--4 | NA |
| HPFS | 0--2.18 | 2.18--3.215 | 3.215--4.4125 | 4.4125-13.18 | NA | NA | NA | NA |
| NHS | NA | NA | NA | NA | 0--2.32 | 2.32--3.25 | 3.25--4.51 | 4.51--14.07 |
| VITAL | 0.13--1.0575 | 1.0575-1.725 | 1.725--2.5 | 2.5--7.83 | 0.14--1.6125 | 1.6125--2.35 | 2.35--2.9325 | 2.9325--7.2 |
| PMH-CCFR | NA | NA | NA | NA | 0.1--1 | 1--1.145 | 1.145--2 | 2—5 |
| **Fruit** | | | | | | | | |
| CCFR | 0.03--0.71 | 0.71--1 | 1—2 | 2--12 | NA | 0.03--2 | 2--8 | NA |
| OFCCR | 0.03--0.71 | 0.71--1 | 1—2 | 2--8 | 0.03--1 | 1--2 | 2--3 | 3—6 |
| DALS | 0--0.93 | 0.93--1.67 | 1.67--2.875 | 2.875--14.64 | 0--1.17 | 1.17--1.94 | 1.94--2.895 | 2.895—20 |
| PLCO | 0--1.9575 | 1.9575-2.795 | 2.795--4.37 | 4.37--19.76 | 0.24--2.2975 | 2.2975--3.22 | 3.22--4.38 | 4.38--14.32 |
| WHI | NA | NA | NA | NA | 0.03--1 | 1--1.78 | 1.78--2.76 | 2.76—8 |
| DACHS | NA | 0--1 | 1—2 | NA | NA | 0--1 | 1--2 | NA |
| HPFS | 0--1.5775 | 1.5775-2.405 | 2.405--3.3225 | 3.3225-13.59 | NA | NA | NA | NA |
| NHS | NA | NA | NA | NA | 0--1.66 | 1.66--2.32 | 2.32--3.2 | 3.2--15.65 |
| VITAL | 0--0.895 | 0.895--1.59 | 1.59--2.4325 | 2.4325--11.7 | 0.09--1.1675 | 1.1675--2.085 | 2.085--2.8375 | 2.8375--8.67 |
| PMH-CCFR | NA | NA | NA | NA | NA | 0.1--1 | 1--5 | NA |
| **Fiber** | | | | | | | | |
| DALS | 4.6--18.855 | 18.855-25.25 | 25.25--31.51 | 31.51—80 | 4--15.55 | 15.55--20.35 | 20.35--26.415 | 26.415--80 |
| PLCO | 6.32--18.49 | 18.49-23.855 | 23.855-29.8125 | 29.813-74.62 | 4.13--15.97 | 15.97--20.06 | 20.06--26.26 | 26.26--45.75 |
| WHI | NA | NA | NA | NA | 1.77--11.19 | 11.19--15.72 | 15.72--20.425 | 20.425--75.43 |
| HPFS | 6.07--18.42 | 18.42--22.17 | 22.17--27.53 | 27.53--52.12 | NA | NA | NA | NA |
| NHS | NA | NA | NA | NA | 3.4--15.1 | 15.1--18 | 18--21.3 | 21.3--68 |
| VITAL | 5.83-15.268 | 15.268-19.88 | 19.88--26.315 | 26.315-57.69 | 4.41-10.5125 | 10.5125-14.515 | 14.515-18.643 | 18.6425-42.07 |

NA, not available as the variable was not assessed in the study or study was conducted in men or women only
