# Supplementary material for: Genome-Wide Diet-Gene Interaction Analyses for Risk of Colorectal Cancer
Source: PLoS Genet. 2014 Apr 17;10(4):e1004228. doi: 10.1371/journal.pgen.1004228 (PMC3990510; doi:10.1371/journal.pgen.1004228)
Supplement: Table S4 — Interaction between rs4143094 and processed meat intake for risk of colorectal cancer based on one common reference group and stratified analysis by genotype (last row) and by quartiles of processed meat (last column). (DOCX) [file pgen.1004228.s005.docx]

**Table S4: Interaction between rs4143094 and processed meat intake for risk of colorectal cancer based on one common reference group and stratified analysis by genotype (last row) and by quartiles of processed meat (last column)**

|  |  | **rs4143094 genotype** | | | | | **OR(95% CI) per T allele within quartiles of processed meat intake** |
| --- | --- | --- | --- | --- | --- | --- | --- |
| **Processed meat intake** | **GG** | | **TG** | | **TT** | |  |
| **in quartiles** | **N Ca/Co** | **OR(95% CI)** | **N Ca/Co** | **OR(95% CI)** | **N Ca/Co** | **OR(95% CI)** |  |
| **1** | 806/865 | 1 | 479/657.4 | 0.78 (0.67-0.92) | 77/119 | 0.68 (0.50-0.92) | 0.80 (0.71-0.90) |
|  |  |  |  | P= 2.1e-03 |  | P= 0.012 | P= 2.7e-04 |
| **2** | 1200/1432 | 0.98 (0.87-1.12) | 803/941 | 1.02 (0.89-1.18) | 146/179 | 1.00 (0.78-1.28) | 1.04 (0.94-1.14) |
|  |  | P= 0.82 |  | P= 0.77 |  | P= 1.00 | P= 0.43 |
| **3** | 894/915 | 1.12 (0.97-1.28) | 593/557 | 1.21 (1.03-1.41) | 94/78 | 1.37 (0.99-1.89) | 1.09 (0.96-1.23) |
|  |  | P= 0.12 |  | P= 0.018 |  | P= 0.054 | P= 0.18 |
| **4** | 727/774 | 1.03 (0.89-1.20) | 553/455 | 1.35 (1.14-1.58) | 113/69 | 1.82 (1.32-2.52) | 1.31 (1.15-1.49) |
|  |  | P= 0.65 |  | P= 3.7e-04 |  | P= 2.8e-04 | P= 3.7e-05 |
| **ORs (95% CI) per quartile processed meat within each genotype** | 3627/3986 | 1.03 (0.98-1.07) | 2428/2610 | 1.20 (1.13-1.26) | 430/445 | 1.39 (1.22-1.59) |  |
|  |  | P= 0.26 |  | P= 2.7e-10 |  | P= 1.1e-06 |  |
