# Supplementary material for: Genome-Wide Diet-Gene Interaction Analyses for Risk of Colorectal Cancer
Source: PLoS Genet. 2014 Apr 17;10(4):e1004228. doi: 10.1371/journal.pgen.1004228 (PMC3990510; doi:10.1371/journal.pgen.1004228)
Supplement: Table S5 — Top three most significant GxE interactions for red meat, vegetable, fruit and fiber using conventional case-control logistic regression analyses (for regions with multiple highly correlated SNPs only the most significant SNP was included). (DOCX) [file pgen.1004228.s006.docx]

**Table S5: Top three most significant GxE interaction for red meat, vegetable, fruit and fiber using conventional case-control logistic regression analyses** (for regions with multiple highly correlated SNPs only the most significant SNP was included)

| **SNP** | **Chr** | **Position** | **Gene** | **CAF** | **OR_interaction_** | **95% CI** | **p_interaction_** | **p_het_** | **Imputed R2** |
| --- | --- | --- | --- | --- | --- | --- | --- | --- | --- |
| **Red meat** | | | | | | | | | |
| rs2901879 | 2 | 66819570 | near MEIS1/DNMT3AP1 | 0.41-0.49 | 0.9 | 0.86-0.94 | 7.30E-07 | 0.2 | 0.82-1.00 |
| rs1871438 | 15 | 90660885 | near NPM1P5/ST8SIA2 | 0.51-0.56 | 1.11 | 1.07-1.16 | 7.56E-07 | 0.17 | 0.83-1.00 |
| rs1370916 | 7 | 47205232 | near MRPL42P4/TNS3 | 0.46-0.52 | 1.11 | 1.06-1.15 | 2.12E-06 | 0.61 | 0.93-0.98 |
| **Vegetable** | | | | | | | | | |
| rs4849303 | 2 | 111444953 | in ACOXL | 0.47-0.54 | 0.89 | 0.85-0.93 | 1.10E-06 | 0.74 | 0.93-1.00 |
| rs4147045 | 4 | 18404428 | near LCORL/RPL21P46 | 0.08-0.12 | 0.83 | 0.77-0.90 | 1.18E-06 | 0.48 | 0.95-0.95 |
| rs6989010 | 8 | 12817126 | near LONRF1/KIAA1456 | 0.98-0.99 | 0.61 | 0.50-0.75 | 1.73E-06 | 0.14 | 0.45-0.98 |
| **Fruit** | | | | | | | | | |
| rs7999699 | 13 | 47267731 | near RPL27AP8/SUCLA2 | 0.38-0.47 | 0.76 | 0.68-0.85 | 2.78E-06 | 0.74 | All genotyped |
| rs11671104 | 19 | 22586526 | near VN1R86P/ZNF492 | 0.89-0.94 | 1.25 | 1.13-1.37 | 4.06E-06 | 0.49 | 0.60-0.95 |
| rs7248888 | 19 | 51665843 | in PNMAL1 | 0.93-0.97 | 0.73 | 0.64-0.84 | 4.90E-06 | 0.51 | 0.47-1.00 |
| **Fiber** | | | | | | | | | |
| rs2593957 | 3 | 110185715 | MORC1 | 0.40-0.46 | 0.87 | 0.83-0.92 | 1.55E-06 | 0.60 | 0.94-1.00 |
| rs12534701 | 7 | 154311843 | DPP6 | 0.54-0.62 | 1.17 | 1.09-1.24 | 1.76E-06 | 0.89 | 0.46-1.00 |
| rs4855695 | 3 | 110334174 | MORC1, FLJ22763 | 0.51-0.58 | 1.14 | 1.08-1.20 | 2.08E-06 | 0.79 | 0.97-1.00 |

CAF, count allele frequency
