# Supplementary material for: Genome-Wide Diet-Gene Interaction Analyses for Risk of Colorectal Cancer
Source: PLoS Genet. 2014 Apr 17;10(4):e1004228. doi: 10.1371/journal.pgen.1004228 (PMC3990510; doi:10.1371/journal.pgen.1004228)
Supplement: Table S6 — Description of bioinformatics tools used for functional follow-up of non-coding regions. (DOCX) [file pgen.1004228.s007.docx]

**Table S6. Description of bioinformatics tools used for functional follow-up of non-coding regions**

| **Dataset** | **Genomic class** | **Description** | **Data source/program** |
| --- | --- | --- | --- |
| 1 | Non-synonymous coding | Exonic positions wherein the variant would cause an amino acid replacement | dbSNP version 131 |
| 2 | Promoter | 1kb regions upstream of annotated transcription start sites | RefSeq |
| 3 | TFBS | Transcription factor binding sites (TFBS) predicted in promoter & non-promoter regulatory elements | UCSC Table Browser [[82](#_ENREF_82)]: ChIPseq Transcription factor [[83](#_ENREF_83)] PWM-scan^a^ JASPAR, CONSITE^,^ HaploReg |
| 4 | Non-coding RNA | All types of experimentally supported non-coding RNA, including microRNAs | RNAdb 2.0 [[84](#_ENREF_84)] & miRBase 17.0 [[85](#_ENREF_85)] |
| 5 | MicroRNA target site | Computationally predicted microRNA target sites within 3’ UTRs | TargetScanS 5.2 [[86](#_ENREF_86)] |
| 6 | Enhancer element | Experimentally supported enhancer elements in any tissue | VISTA Enhancer Browser  UCSC Table Browser [[82](#_ENREF_82)]: ENCODE ChIP-seq Histone Modification [[83](#_ENREF_83)] |
| 7 | Candidate non-specific regulatory element | Open chromatin loci in at least one human cell type, as assessed by DNase I hypersensitivity (DHS) mapping | UCSC Table Browser [[82](#_ENREF_82)]: Duke and UW DNase I HS data from > 50 cell types [[83](#_ENREF_83)] |
| 8 | Insulator elements | CTCF binding sites assessed by ChIP-seq technology | UCSC Table Browser [[82](#_ENREF_82)]: ChIP-seq TFBS [[83](#_ENREF_83)] |
| 9 | eQTL | Allele-specific differences in expression levels | GTEx eQTL Browser [[87](#_ENREF_87)] |
| 10 | Conserved Element |  | UCSC Table Browser [[82](#_ENREF_82)]: PhastCons 46-way conservation [[88](#_ENREF_88)] |
| 11 | Splice Site |  | BDGP |
| ^a^PWM-scan was applied using positional weight matrices (PWMs) from the Transfac database | | | |
