# Supplementary material for: Genome-Wide Diet-Gene Interaction Analyses for Risk of Colorectal Cancer
Source: PLoS Genet. 2014 Apr 17;10(4):e1004228. doi: 10.1371/journal.pgen.1004228 (PMC3990510; doi:10.1371/journal.pgen.1004228)
Supplement: Text S1 — Study populations. Description of the methodology and individual study populations included in this meta-analysis. (DOCX) [file pgen.1004228.s008.docx]

**Description of Study Populations**

In the following we describe each study population used in the genome-wide interaction analysis. For information on sample sizes and demographic factors please see Supplemental Table 1.

***Colon Cancer Family Registry (CCFR)***. The CCFR is an NCI-supported consortium consisting of six centers dedicated to the establishment of a comprehensive collaborative infrastructure for interdisciplinary studies in the genetic epidemiology of colorectal cancer [[39](#_ENREF_39)]. The CCFR includes data from approximately 30,500 total subjects (10,500 probands, and 20,000 unaffected and affected relatives and unrelated controls). Cases and controls, age 20 to 74 years, were recruited at the six participating centers beginning in 1998. CCFR implemented a standardized questionnaire that is administered to all participants, and includes established and suspected risk factors for colorectal cancer, which includes questions on medical history and medication use, reproductive history (for female participants), family history, physical activity, demographics, alcohol and tobacco use, and dietary factors. For genome-wide interaction analysis the CCFR scan has been described previously [[13](#_ENREF_13)], includes population-based cases and age-matched controls from the three population-based centers: Seattle, Toronto and Australia. Cases were genetically enriched by over-sampling those with a young age at onset or positive family history. Controls were matched to cases on age and sex. All cases and controls were self-reported as White, which was confirmed with genotype data.

***Darmkrebs: Chancen der Verhütung durch Screening (DACHS)*** [[45](#_ENREF_45),[65](#_ENREF_65)]. This German study was initiated as a large population-based case-control study in 2003 in the Rhine-Neckar-Odenwald region (southwest region of Germany) to assess the potential of endoscopic screening for reduction of colorectal cancer risk and to investigate etiologic determinants of disease, particularly lifestyle/environmental factors and genetic factors. Cases with a first diagnosis of invasive colorectal cancer (ICO-10 codes C18-C20) who were at least 30 years of age (no upper age limit), German speaking, a resident in the study region, and mentally and physically able to participate in a one-hour interview, were recruited by their treating physicians either in the hospital a few days after surgery, or by mail after discharge from the hospital. Cases were confirmed based on histologic reports and hospital discharge letters following diagnosis of colorectal cancer. All hospitals treating colorectal cancer patients in the study region participated. Based on estimates from population-based cancer registries, more than 50% of all potentially eligible patients with incident colorectal cancer in the study region were included. Community-based controls were randomly selected from population registries, employing frequency matching with respect to age (5-year groups), sex, and county of residence. Controls with a history of colorectal cancer were excluded. Controls were contacted by mail and follow-up calls. The participation rate was 51%. During an in-person interview, data were collected on demographics, medical history, family history of CRC, and various life-style factors, as were blood and mouthwash samples. The Set 1 scan consisted of a subset of participants recruited up to 2007, and samples were frequency matched on age and gender. The Set 2 scan consisted of additional subjects that were recruited up to 2010 as part of this ongoing study.

***Diet, Activity, and Lifestyle Study (DALS****)* [[40](#_ENREF_40)]. DALS is a population-based case-control study of colon cancer. Participants were recruited between 1991 and 1994 from three locations: the Kaiser Permanente Medical Care Program (KPMCP) of Northern California, an eight-county area in Utah, and the metropolitan Twin Cities area of Minnesota. Eligibility criteria for cases included age at diagnosis between 30 and 79 years, diagnosis with first primary colon cancer (ICD-O-2 codes 18.0 and 18.2-18.9) between October 1^st^ 1991 and September 30^th^ 1994, English speaking, and competency to complete the interview. Individuals with cancer of the rectosigmoid junction or rectum were excluded, as were those with a pathology report noting familial adenomatous polyposis, Crohn’s disease, or ulcerative colitis. A rapid-reporting system was used to identify all incident cases of colon cancer resulting in the majority of cases being interviewed within four months of diagnosis. Controls from KPMCP were randomly selected from membership lists. In Utah, controls under 65 years of age were randomly selected through random-digit dialing and driver license lists. Controls, 65 years of age and older, were randomly selected from Health Care Financing Administration lists. In Minnesota, controls were identified from Minnesota driver’s license or state ID lists. Cases and controls were matched to cases by 5-year age groups and sex. The Set I scan consisted of a subset of the study designed above, from Utah, Minnesota, and KPMCP, and was restricted to subjects who self-reported as White non-Hispanic. The Set 2 scan consisted of subjects from Utah and Minnesota that were not genotyped in Set 1. Set 2 was restricted to subjects who self-reported as White non-Hispanic and those that had appropriate consent to post data to dbGaP.

***Health Professionals Follow-up Study (HPFS)*** [[66](#_ENREF_66)]. The HPFS is a parallel prospective study to the Nurses’ Health Study (NHS). The HPFS cohort comprises 51,529 men who, in 1986, responded to a mailed questionnaire. The participants are U.S. male dentists, optometrists, osteopaths, podiatrists, pharmacists, and veterinarians born between 1910 and 1946. Participants have provided information on health related exposures, including: current and past smoking history, age, weight, height, diet, physical activity, aspirin use, and family history of colorectal cancer. Colorectal cancer and other outcomes were reported by participants or next-of-kin and followed up through review of the medical and pathology record by physicians. Overall, more than 97% of self-reported colorectal cancers were confirmed by medical record review. Information was abstracted on histology and primary location.. Follow-up has been excellent, with 94% of the men responding to date. Colorectal cancer cases were ascertained through January 1, 2008. In 1993-95, 18,825 men in HPFS mailed in blood samples by overnight courier which were aliquoted into buffy coat and stored in liquid nitrogen. In 2001-04, 13,956 men in HPFS who had not previously provided a blood sample mailed in a "swish-and-spit" sample of buccal cells. Incident cases are defined as those occurring after the subject provided a blood or buccal sample. Prevalent cases are defined as those occurring after enrollment in the study in 1986, but prior to the subject providing either a blood or buccal sample. All lifestyle and dietary data for these analyses are based on information collected prior to diagnosis. After excluding participants with histories of cancer (except non-melanoma skin), ulcerative colitis, or familial polyposis, two case-control sets were constructed from which DNA was isolated from either buffy coat or buccal cells for genotyping: 1) a case-control set with cases of colorectal cancer matched to randomly selected controls who provided a blood sample and were free of colorectal cancer at the same time the colorectal cancer was diagnosed in the cases; 2) a case-control set with cases of colorectal cancer matched to randomly selected controls who provided a buccal sample and were free of colorectal cancer at the same time the colorectal cancer was diagnosed in the case. For both case-control sets, matching criteria included year of birth (within 1 year) and month/year of blood or buccal cell sampling (within six months). Cases were pair matched 1:1, 1:2, or 1:3 with a control participant(s).

***Nurses’ Health Study (NHS)*** [[67](#_ENREF_67)]. The NHS cohort began in 1976 when 121,700 married female registered nurses aged 30 to 55 years returned the initial questionnaire that ascertained a variety of important health-related exposures. Since 1976, follow-up questionnaires have been mailed every two years. Colorectal cancer and other outcomes were reported by participants or next-of-kin and followed up through review of the medical and pathology record by physicians. Overall, more than 97% of self-reported colorectal cancers were confirmed by medical-record review. Information was abstracted on histology and primary location. Follow-up has been high: as a proportion of the total possible follow-up time, follow-up has been over 92%. Colorectal cancer cases were ascertained through June 1, 2008. In 1989-90, 32,826 women in NHS I, mailed in blood samples by overnight courier which were aliquoted into buffy coat and stored in liquid nitrogen. In 2001-04, 29,684 women in NHS I who did not previously provide a blood sample mailed in a "swish-and-spit" sample of buccal cells. Incident cases are defined as those occurring after the subject provided a blood or buccal sample. Prevalent cases are defined as those occurring after enrollment in the study in 1976, but prior to the subject providing either a blood or buccal sample. All lifestyle and dietary data for these analyses are based on information collected prior to diagnosis. After excluding participants with histories of cancer (except non-melanoma skin), ulcerative colitis, or familial polyposis, we constructed two case-control sets from which DNA was isolated from either buffy coat or buccal cells for genotyping: 1) a case-control set with cases of colorectal cancer matched to randomly selected controls who provided a blood sample and were free of colorectal cancer at the same time the colorectal cancer was diagnosed in the case; 2) a case-control set with cases of colorectal cancer matched to randomly selected controls who provided a buccal sample and were free of colorectal cancer at the same time the colorectal cancer was diagnosed in the cases. For both case-control sets, matching criteria included year of birth (within one year) and month / year of blood or buccal cell sampling (within six months). Cases were pair matched 1:1, 1:2, or 1:3 with a control participant(s).

***Ontario Familial Colorectal Cancer Registry*** *(****OFCCR)***. A subset of the Assessment of Risk in Colorectal Tumours in Canada (ARCTIC) from the Ontario Registry for Studies of Familial Colorectal Cancer (OFCCR) was used. Both the case-control study [[68](#_ENREF_68)] and the OFCCR [[69](#_ENREF_69)] have been described in detail previously, as have GWAS results [[22](#_ENREF_22)]. In brief, cases were confirmed incident colorectal cancer (CRC) cases ages 20 to 74 years, residents of Ontario identified through comprehensive registry and diagnosed between July 1997 and June 2000. Population-based controls were randomly selected among Ontario residents (random-digit-dialing and listing of all Ontario residents), and matched by sex and 5-year age groups. A total of 1,236 CRC cases and 1,223 controls were successfully genotyped on at least one of the Illumina 1536 GoldenGate assay, the Affymetrix GeneChip® Human Mapping 100K and 500K Array Set, and a 10K non-synonymous SNP chip. Analysis was based on a set of unrelated subjects who were non-Hispanic, White by self-report or by investigation of genetic ancestry. We further excluded subjects if there was a sample mix-up, if they were missing epidemiologic questionnaire data, if they were appendix cases, or if they were overlapped with the Colon Cancer Family Registry GWAS (see above). Additionally, only samples genotyped on the Affymetrix GeneChip® 500K Array were utilized in order to avoid coverage issues in imputation.

***Prostate, Lung, Colorectal, and Ovarian Cancer Screening Trial (PLCO)***. PLCO enrolled 154,934 participants (men and women, aged between 55 and 74 years) at ten centers into a large, randomized, two-arm trial to determine the effectiveness of screening to reduce cancer mortality. Sequential blood samples were collected from participants assigned to the screening arm. Participation was 93% at the baseline blood draw. In the observational (control) arm, buccal cells were collected via mail using the “swish-and-spit” protocol and participation rate was 65%. Details of this study have been previously described [[42](#_ENREF_42),[70](#_ENREF_70)] and are available online (http://dcp.cancer.gov/plco).

The Set 1 GWAS included a subset of 560 colon cancer cases self-reported as being non-Hispanic White with available DNA samples, questionnaire data, and appropriate consent for ancillary epidemiologic studies. Cases had been excluded from selection if they had a history of inflammatory bowel disease, polyps, polyposis syndrome or cancer (excluding basal or squamous cell skin cancer). Sex- and age matched controls were selected among participants from the Cancer Genetic Markers of Susceptibility (CGEMS) prostate cancer scan [[71](#_ENREF_71),[72](#_ENREF_72)] and the GWAS of Lung Cancer and Smoking [[73](#_ENREF_73)] and the Pancreatic Cancer Cohort Consortium (PanScan) [[74](#_ENREF_74),[75](#_ENREF_75)] along with an additional 92 non-Hispanic White female controls that were genotyped together with the cases. For the Set 2 scan, cases were colorectal cancers from both arms of the trial, which were not already included in Set 1. Samples were excluded if participants did not sign appropriate consents, if DNA was unavailable, if baseline questionnaire data with follow-up were unavailable, if they had a history of colon cancer prior to the trial, if they were a rare cancer, and if they were already in colon GWAS, or if they were a control in the prostate or lung populations. Controls were frequency matched 1:1 to cases without replacement, and cases were not eligible to be controls. Matching criteria were age at enrollment (two year blocks), enrollment date (two year blocks), sex, race / ethnicity, trial arm, and study year of diagnosis (i.e. controls must be cancer free into the case's year of diagnosis).

***Postmenopausal Hormones Supplementary Study to the Colon Cancer Family Registry (PMH-CCFR*)** [[76](#_ENREF_76)]. Eligible case patients included all female residents, ages 50 to 74 years, residing in the 13 counties in Washington State reporting to the Cancer Surveillance SEER program, who were newly diagnosed with invasive colorectal adenocarcinoma (ICD-O C18.0, C18.2-.9, C19.9, C20.0-.9) between October 1998 and February 2002. Eligibility for all individuals was limited to those who were English-speaking with available telephone numbers, in which they could be contacted. On average, cases were identified within four months of diagnosis. The overall response proportion of eligible cases identified was 73%. Community-based controls were randomly selected according to age distribution (in 5-year age intervals) of the eligible cases by using lists of licensed drivers from the Washington State Department of Licensing for individuals, ages 50 to 64 years, and rosters from the Health Care Financing Administration (now the Centers for Medicare and Medicaid) for individuals older than 64 years. The overall response proportion of eligible controls was 66%. In GECCO, samples with sufficient DNA extracted from blood were genotyped. Only participants that were not part of the CCFR Seattle site were included in the sample set.

***VITamins And Lifestyle (VITAL).*** The VITamins And Lifestyle (VITAL) cohort comprises of 77,721 Washington State men and women aged 50 to 76 years, recruited from 2000 to 2002 to investigate the association of supplement use and lifestyle factors with cancer risk. Subjects were recruited by mail, from October 2000 to December 2002, using names purchased from a commercial mailing list. All subjects competed a 24 page questionnaire and buccal-cell specimens for DNA was self-collected by 70% of the participants. Subjects are followed for cancer by linkage to the western Washington SEER cancer registry and are censored when they move out of the area covered by the registry or at time of death. Details of this study have been previously described [[77](#_ENREF_77)]. In GECCO, a nested case-control set was genotyped. Samples included, colorectal cancer cases with DNA, excluding subject with colorectal cancer before baseline, in situ cases, (large cell) neuroendocrine carcinoma, squamous cell carcinoma, carcinoid tumor, Goblet cell carcinoid, any type of lymphoma, including non-Hodgkin, Mantle cell, large B-cell, or follicular lymphoma. Controls were matched on age at enrollment (within one year), enrollment date (within one year), sex, and race / ethnicity. One control was randomly selected per case among all controls that matched on the four factors above and where the control follow-up time was greater than follow-up time of the case until diagnosis.

**Women’s Health Initiative (WHI).** WHI is a long-term health study of 161,808 post-menopausal women aged 50 to 79 years at 40 clinical centers throughout the U.S. WHI comprises a Clinical Trial (CT) arm, an Observational Study (OS) arm, and several extension studies. The details of WHI have been previously described [[78](#_ENREF_78),[79](#_ENREF_79)] and are available online (https://cleo.whi.org/SitePages/Home.aspx). In GECCO, Set 1 cases were selected from the September 12, 2005 database and were comprised of centrally adjudicated colon cancer cases from the Observational Study (OS) who self-reported as White. Controls were first selected among controls previously genotyped as part of a Hip Fracture GWAS conducted within the WHI OS and matched to cases on age (within three years) enrollment date (within 365 days), hysterectomy status, and prevalent conditions at baseline. For 37 cases, there was not a control match in the Hip Fracture GWAS. For these participants, we identified a matched control in the WHI OS based on same criteria. In the Set 2 scan, cases were selected from the August 2009 database and were comprised of centrally adjudicated colon and colorectal cancer cases from the OS and CT who were not genotyped in Set 1. In addition, case and control participants were subject to the following exclusion criteria: a prior history of colorectal cancer at baseline, IRB approval not available for data submission into dbGaP, and not sufficient DNA available. Matching criteria included age (within years), race/ethnicity, WHI date (within three years), WHI Calcium and Vitamin D study date (within three years), and randomization arms (OS flag, hormone therapy assignments, dietary modification assignments, calcium/vitamin D assignments). In addition, they were matched on the four regions of randomization centers. Each case was matched with one control (1:1) that exactly met the matching criteria. Control selection was done in a time-forward manner, selecting one control for each case first from the risk set at the time of the case’s event. The matching algorithm was allowed to select the closest match based on a criterion to minimize an overall distance measure [[80](#_ENREF_80)]. Each matching factor was given the same weight. Additional controls that were genotyped as part of the Hip Fracture GWAS were included to improve power.
