# Supplementary material for: Genome-Wide Diet-Gene Interaction Analyses for Risk of Colorectal Cancer
Source: PLoS Genet. 2014 Apr 17;10(4):e1004228. doi: 10.1371/journal.pgen.1004228 (PMC3990510; doi:10.1371/journal.pgen.1004228)
Supplement: Text S2 — Additional statistical analysis. Description of the additional statistical methods used in this meta-analysis. (DOCX) [file pgen.1004228.s009.docx]

**Additional Statistical Analysis Methods**

The Gauderman et al. method [[16](#_ENREF_16)] consists of two steps: screening and testing for GxE. The screening statistic in Gauderman et al’s method is defined as the sum of the square of z statistics for marginal association of G with disease risk and correlation of G with E [[59](#_ENREF_59)]. Because this combined screening statistic included correlation screening, we used only the CC test for GxE interaction to avoid inflating type I error. Weighted hypothesis testing was used for multiple comparison adjustment consistent with the Cocktail method [[15](#_ENREF_15)].

A 2-degrees-of-freedom (2 d. f.) chi-squared test was also performed to jointly test marginal association of G and GxE interaction to identify SNPs that affect CRC risk, exploiting possible heterogeneous SNP effects in different levels of D [[81](#_ENREF_81)]. This was done by taking the sum of the square of the z statistic for marginal association of G with disease risk and the square of the z statistic from the case-only analysis of GxE interaction, and the p-value was determined by comparing the sum with a 2 d.f. chisquare distribution [[17](#_ENREF_17)].

To estimate the effects of environment variable stratified by genotypes, we fit the following model:


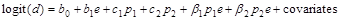
, where
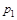
 and
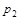
 are the imputation posterior probabilities for genotype A/B, B/B. Then the stratified effects of environment variable can be estimated as
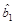
,
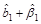
,
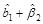
 for genotype A/A, A/B, and B/B, respectively and the standard errors can be estimated by using the standard formula for linear combination of two parameters based on the covariance matrix of these parameters.
