# Supplementary material for: Genome-Wide Diet-Gene Interaction Analyses for Risk of Colorectal Cancer
Source: PLoS Genet. 2014 Apr 17;10(4):e1004228. doi: 10.1371/journal.pgen.1004228 (PMC3990510; doi:10.1371/journal.pgen.1004228)
Supplement: Text S3 — Functional annotation of identified loci. Description of the methodology for functionally annotating significant loci. (DOCX) [file pgen.1004228.s010.docx]

**Functional annotation of identified loci**

We conducted a functional annotation for SNPs (and correlated SNPs) that showed a significant interaction. As detailed in the Text S3 and Table S6, we queried multiple bioinformatic databases using the University of California, Santa Cruz genome browser and the Broad Institutes database, HaploReg.
