# Supplementary material for: Genome-Wide Diet-Gene Interaction Analyses for Risk of Colorectal Cancer
Source: PLoS Genet. 2014 Apr 17;10(4):e1004228. doi: 10.1371/journal.pgen.1004228 (PMC3990510; doi:10.1371/journal.pgen.1004228)
Supplement: Text S4 — References supplementary text. (DOCX) [file pgen.1004228.s011.docx]

**Additional References:**

65. Lilla C, Verla-Tebit E, Risch A, Jager B, Hoffmeister M, et al. (2006) Effect of NAT1 and NAT2 genetic polymorphisms on colorectal cancer risk associated with exposure to tobacco smoke and meat consumption. Cancer epidemiology, biomarkers & prevention : a publication of the American Association for Cancer Research, cosponsored by the American Society of Preventive Oncology 15: 99-107.

66. Rimm EB, Stampfer MJ, Colditz GA, Chute CG, Litin LB, et al. (1990) Validity of self-reported waist and hip circumferences in men and women. Epidemiology 1: 466-473.

67. Belanger CF, Hennekens CH, Rosner B, Speizer FE (1978) The nurses' health study. Am J Nurs 78: 1039-1040.

68. Cotterchio M, Manno M, Klar N, McLaughlin J, Gallinger S (2005) Colorectal screening is associated with reduced colorectal cancer risk: a case-control study within the population-based Ontario Familial Colorectal Cancer Registry. Cancer causes & control : CCC 16: 865-875.

69. Cotterchio M, McKeown-Eyssen G, Sutherland H, Buchan G, Aronson M, et al. (2000) Ontario familial colon cancer registry: methods and first-year response rates. Chronic Dis Can 21: 81-86.

70. Gohagan JK, Prorok PC, Hayes RB, Kramer BS (2000) The Prostate, Lung, Colorectal and Ovarian (PLCO) Cancer Screening Trial of the National Cancer Institute: history, organization, and status. Controlled clinical trials 21: 251S-272S.

71. (2009) National Cancer Institute. Cancer Genetic Markers of Susceptibility (CGEMS) data website.

72. Yeager M, Orr N, Hayes RB, Jacobs KB, Kraft P, et al. (2007) Genome-wide association study of prostate cancer identifies a second risk locus at 8q24. Nat Genet 39: 645-649.

73. Landi MT, Chatterjee N, Yu K, Goldin LR, Goldstein AM, et al. (2009) A genome-wide association study of lung cancer identifies a region of chromosome 5p15 associated with risk for adenocarcinoma. American journal of human genetics 85: 679-691.

74. Amundadottir L, Kraft P, Stolzenberg-Solomon RZ, Fuchs CS, Petersen GM, et al. (2009) Genome-wide association study identifies variants in the ABO locus associated with susceptibility to pancreatic cancer. Nat Genet 41: 986-990.

75. Petersen GM, Amundadottir L, Fuchs CS, Kraft P, Stolzenberg-Solomon RZ, et al. (2010) A genome-wide association study identifies pancreatic cancer susceptibility loci on chromosomes 13q22.1, 1q32.1 and 5p15.33. Nat Genet 42: 224-228.

76. Newcomb PA, Zheng Y, Chia VM, Morimoto LM, Doria-Rose VP, et al. (2007) Estrogen plus progestin use, microsatellite instability, and the risk of colorectal cancer in women. Cancer research 67: 7534-7539.

77. White E, Patterson RE, Kristal AR, Thornquist M, King I, et al. (2004) VITamins And Lifestyle cohort study: study design and characteristics of supplement users. American journal of epidemiology 159: 83-93.

78. Hays J, Hunt JR, Hubbell FA, Anderson GL, Limacher M, et al. (2003) The Women's Health Initiative recruitment methods and results. Ann Epidemiol 13: S18-77.

79. (1998) Design of the Women's Health Initiative clinical trial and observational study. The Women's Health Initiative Study Group. Controlled clinical trials 19: 61-109.

80. Bergstralh EJ, Kosanke JL (1995) Computerized matching of cases to controls. 56 ed. ; Department of Health Sciences Research MC, editor. Rochester MN.

81. Kraft P, Yen YC, Stram DO, Morrison J, Gauderman WJ (2007) Exploiting gene-environment interaction to detect genetic associations. Human heredity 63: 111-119.
